# Compositional Data Analysis of 16S rRNA Gene Sequencing Results from Hospital Airborne Microbiome Samples

**DOI:** 10.3390/ijerph191610107

**Published:** 2022-08-16

**Authors:** Maria Rita Perrone, Salvatore Romano, Giuseppe De Maria, Paolo Tundo, Anna Rita Bruno, Luigi Tagliaferro, Michele Maffia, Mattia Fragola

**Affiliations:** 1 Department of Mathematics and Physics, University of Salento, 73100 Lecce, Italy; 2 Presidio Ospedaliero Santa Caterina Novella, Azienda Sanitaria Locale Lecce, 73013 Galatina, Italy; 3 Department of Biological and Environmental Sciences and Technologies, University of Salento, 73100 Lecce, Italy

**Keywords:** 16S rRNA gene sequencing, Aitchison distance, CLR transformation, singular value decomposition, alpha-diversity, airborne microbiome, compositional data, ρ metrics

## Abstract

The compositional analysis of 16S rRNA gene sequencing datasets is applied to characterize the bacterial structure of airborne samples collected in different locations of a hospital infection disease department hosting COVID-19 patients, as well as to investigate the relationships among bacterial taxa at the genus and species level. The exploration of the centered log-ratio transformed data by the principal component analysis via the singular value decomposition has shown that the collected samples segregated with an observable separation depending on the monitoring location. More specifically, two main sample clusters were identified with regards to bacterial genera (species), consisting of samples mostly collected in rooms with and without COVID-19 patients, respectively. Human pathogenic genera (species) associated with nosocomial infections were mostly found in samples from areas hosting patients, while non-pathogenic genera (species) mainly isolated from soil were detected in the other samples. *Propionibacterium acnes*, *Staphylococcus pettenkoferi*, *Corynebacterium tuberculostearicum*, and *jeikeium* were the main pathogenic species detected in COVID-19 patients’ rooms. Samples from these locations were on average characterized by smaller richness/evenness and diversity than the other ones, both at the genus and species level. Finally, the ρ metrics revealed that pairwise positive associations occurred either between pathogenic or non-pathogenic taxa.

## 1. Introduction

The identification of the most abundant taxa in tested environments represents the main goal of most studies based on microbial communities characterized by DNA sequencing, as the 16S rRNA gene sequencing. Nevertheless, the choice of the best approach to identify which taxa significantly differ in relative abundance between groups of samples is still the subject of controversial debate [1,2,3]. The compositional nature of microbiome data from high-throughput sequencing makes them challenging to explore [4,5], since it does not allow using traditional statistical procedures [1,6]. Aitchison [7] identified compositional data as the ones “that contain information about the relationships between the parts”, while Gloor et al. [2] defined them as those “that are naturally described as proportions or probabilities, or with a constant or irrelevant sum” and showed that the use of standard statistical approaches to analyze compositional data could generate only uninterpretable results. Aitchison realized that each compositional dataset could be rephrased in terms of ratios of components and developed some basic theories and different methods, procedures, and tools for the compositional data analysis [8,9]. The ratio transformation of data represented the starting point for all the other developed compositional approaches since these ratios are independently the same whether the data are counts or proportions. The logarithm of these ratios (defined as log-ratio transformation) generally represented the most common approach accepted by statisticians and many researchers in different fields (e.g., [10,11]). The main advantage of log-ratio transformations is that the problem of a constrained sample space of the compositional data can be removed, and in addition, data are projected into multivariate real space, which allows using all available classic multivariate approaches to examine compositional datasets (e.g., [12]). Sophisticated data analysis concepts and methodologies were developed and used to investigate the compositional microbiome data from high-throughput sequencing results (e.g., [6,12,13,14,15]). In addition to showing that the analysis of compositional data by traditional methods could be misleading and unpredictable, Gloor et al. [2] proposed a compositional approach workflow. The first step of the analysis workflow is represented by a centered log-ratio transformation (CLR) of the input compositional dataset from high-throughput sequencing [2]. Then, they proposed the Aitchison distance (i.e., the Euclidean distance between samples after CLR transformation of OTUs/reads) as a compositional replacement for distance determination to be used for clustering and ordination techniques. Gloor et al. [2] explained that the Aitchison distance is superior to the most common Bray–Curtis dissimilarity metrics, since it is more stable to aggregate the data, in addition to be a true linear distance [16]. Robinson et al. [17] investigated the bacterial beta-diversity for both soil and airborne samples in the southern Adelaide Parklands (Australia) by using ordination plots of Aitchison distances based on CLR-transformations of OTU abundances obtained from 16S rRNA gene sequencing datasets. The third step of the compositional analysis workflow [2] is represented by the variance-based compositional principal component (PCA) biplot [18], as replacement for beta-diversity exploration of microbiome data. The main reason of this last selection is that this kind of PCA biplot can be used to identify the relationships among inter-OTU variance and sample distance [13]. In addition, compositional PCA biplots present many benefits over the principal coordinate analysis (PCoA) plots for beta-diversity analysis. Firstly, the results obtained from compositional PCA biplots are more stable than the ones from the corresponding PCoA starting from the same dataset [19]. Secondly, PCA plots can be more reproducible. More specifically, Gloor et al. [2] suggested the compositional PCA biplot made by a singular value decomposition (SVD) of the CLR-transformed data as the first exploratory tool to be used to examine the dataset [18]. In fact, the SVD-PCA biplot is generally applied to obtain the best least-square representation of the data matrix in a low-dimensional space, showing the Euclidean distances between samples in addition to the variances and correlations of the analyzed variables [20]. Grześkowiak et al. [21] estimated the principal components for their dataset of pig gut microbiota via singular value decomposition of the data matrix as defined by *prcomp* method under the R package *stats*. Many previous studies used the compositional SVD-PCA biplot to analyze their microbiome data (e.g., [15,20,22]). Gloor et al. [2] and Xia et al. [4] have also provided a detailed explanation on the inappropriateness of the traditional correlation parameters, such as the Spearman and Pearson coefficients in compositional datasets, because of the likely occurrence of spurious correlations, negative correlation biases, false positive correlations, and instability to subset the data. Therefore, new statistically rigorous methods, such as SPARCC [23] and SPieCeasi [24], were developed to perform a correlation analysis for microbiome datasets in case of a sparse data matrix. Gloor et al. [2] suggested the use of the φ [25] or the ρ [26] metrics when the initial microbiome dataset is represented by a non-sparse matrix. These last metrics describe the strength of the proportionality between two variables and do not change whether applied to relative values or to their absolute equivalent values [4]. In more detail, φ and ρ metrics represent the scale log-ratio variance by the variance of one or both constituent parts [27]. Skinnider et al. [28] successfully proved the reliability of both parameters for genomics studies. The R package *propr* allows the calculation of both φ and ρ metrics, in addition to an expected value of the ρ coefficient, generally denoted as E(ρ), which is also valid for sparse data matrix [29]. This expected value E(ρ) assumes a value of 1 if two taxa present exactly constant ratios in the microbiome dataset. Bian et al. [19] determined the E(ρ) value to identify clusters of associated OTUs in their dataset from the analysis of 16S rRNA gene sequencing results for the gut microbiota of a cross-sectional cohort of more than 1000 healthy Chinese individuals. Note that both φ and ρ metrics are sub-compositionally coherent: both these metrics assume the same value for pairs of taxa in common if the whole dataset is analyzed or any subset in the dataset is analyzed [2]. Therefore, the definition of an ideal and common methodology for the characterization of compositional datasets is still an open research challenge, as proved by several recent works (e.g., [30,31,32,33,34,35]). 

In this study, the 16S rRNA gene sequencing results from airborne bioaerosol samples collected at Santa Caterina Novella Hospital in Galatina (Lecce, Italy) have been used to apply the compositional analysis workflow suggested by Gloor et al. [2]. For this purpose, we used the CLR transformation as normalization procedure, the Aitchison distance as basis for data ordination, the compositional SVD-PCA biplot for data clustering, and the ρ metrics for data correlation/proportionality. The analyzed 16S rRNA gene sequencing dataset was extracted from airborne samples collected at different locations of the Infectious Disease (ID) Department of Santa Caterina Novella Hospital when COVID-19 patients were hosted. The airborne bacterial community profiles up to the species level, the main relationships among taxa, and the ones associated with different monitoring locations have been analyzed by the selected compositional analysis approach.

## 2. Materials and Methods

### 2.1. Collection Methodology for Bioaerosol Samples

The lightweight and portable ACD-200 Bobcat, which is a dry-filter and high-volume air sampler (InnovaPrep, Drexel, MO, USA), was used to collect the airborne aerosol and bioaerosol samples analyzed in this study. The device collection medium for ACD-200 Bobcat is a sterile 52-mm-diameter electret filter consisting of electrostatically charged dielectric polymer fibers [36]. Electret filters allow a high collection efficiency of airborne components, including viruses, bacteria, pollen, moulds, and fungal spores, as well as nonbiological particles [37]. The ACD-200 Bobcat allows collecting airborne components from 0.1 to about 10 μm in size with minimal pressure drops, high sampling rates, and long sampling periods [38]. The air sampling occurred at a height of about 50 cm above the floor to minimize contamination, following the recommendations by King et al. [39]. We used sterile latex gloves to insert each electret filter equipped with a filter holder cup (contained in a sterilized box before its use) into the ACD-200 Bobcat air sampler. Once sampling was complete, the filter-holder-cup system was removed from the collector with gloved hands, and a sterile canister containing an elution fluid was inverted over the filter-cup system. More specifically, the sterilized single-use canister contained a wet foam carbon-compressed elution kit patented by the Bobcat’s manufacturer and composed of water, a pH buffer, and a low concentration surfactant (less than 0.1%) infused with carbon dioxide (0.075% Tween 20/PBS). The elution foam is released from the canister through the filter passing through its interstitial spaces to efficiently extract all captured components in 6–7 mL liquid. In fact, the foam immediately collapses back to a liquid phase in the sampling cup, making it available for the following sample processing and analysis phases. More details on the sampling methodology and the main features of ACD-200 Bobcat device can be found in Bøifot et al. [40]. Several recent studies used ACD-200 Bobcat to collect bioaerosol samples in different sampling conditions: vertically distributed across the troposphere [41], near open wastewater canals [42,43], and in farms with swine housing [44,45]. It was also shown that Bobcat is one of the best suited sampling devices to perform indoor bioaerosol collection in hospital wards as in this study (e.g., [39,46,47]), since filter contamination by the operators is unlikely to occur with respect to other commonly used air samplers.

### 2.2. Description of the Sampling Locations

Figure S1 provides a scheme of the ID ward where the indoor samples were collected from 30 April to 4 June 2020, when the ID ward hosted 5 to 9 patients affected by COVID-19. Visitors’ access to the ID ward was entirely restricted during the sampling period. The ID Department is made up of 5 patient rooms with conventional air conditioning systems (denoted as conventional rooms), three high-isolation patient rooms at negative-pressure, the doctor’s office, and the medicine-store room (MED). Twenty-five air changes per hour leading to about 800 m^3^ per hour of outdoor air intake are regularly performed in the high-isolation patient rooms. All patient rooms are equipped with a private bathroom. Table 1 summarizes the acronyms and the sampling dates of all the air samples investigated in this study. Eight and six samples were collected in rooms with and without COVID-19 patients, respectively. A_HR and A_R1 represent the air samples collected in a high isolation room (HR) and in a conventional room (R), respectively, which hosted the COVID-19 patient denoted with the capital letter A. Samples B_R1 and B_R2 were collected in the conventional room that hosted the COVID-19 patient B. B_BAT represents the air sample collected in the bathroom of patient B. B+C_R1 and B+C_R2 are associated with the air samples collected in the conventional room that hosted both the B and C COVID-19 patients. Samples HR1 and HR2 and R3 and R4 were collected in the high isolation rooms 1 and 2, respectively, and the conventional rooms 3 and 4, respectively, without any patients to investigate the relationships between the bacterial profile in rooms with and without COVID-19 patients. Sample MED was collected in the medicine-store room, reserved only to medical staff and healthcare operators. Two electret-filter-holder-cups were used to collect airborne samples by gravimetric dry deposition (DD) over 14 days in the ID Department. More specifically, sample DD1 was collected in a COVID-19 patient room at about 1 m away from the patient’s bed, while sample DD2 was collected in the corridor at about 1 m from the floor. Note that the starting sampling date of samples DD1 and DD2 has been reported in Table 1. Finally, an indoor air sample, denoted as PSY, was collected in a hospitalization room of the psychiatry department, and two outdoor samples (RO1 and RO2) were collected on the roof of the ID Department to investigate the relationships of 16S rRNA gene sequencing results of these last three samples with the ones from the ID Department. Therefore, 14 out of the 17 air samples analyzed in the current study were collected at the ID ward. Bureaucratic problems involving the permission to perform further measurements in the hospital, as well as the lack of COVID-19 patients after the first week of June, limited the amount of the collected samples. 

### 2.3. Methodology for DNA Extraction and 16S rRNA Gene Metabarcoding Approach

Each liquid sample was stored at −30 °C before being treated by means of the DNeasy PowerWater kit (Qiagen, Düsseldorf, Germany) for the DNA extraction. The genomic DNA was extracted following the manufacturer’s suggestions and put in storage at −30 °C for additional examination. The company Genomix4life S.R.L. (Baronissi, Salerno, Italy) performed both the high-throughput sequencing tests and the primary bioinformatics analyses on the liquid samples, following the procedures described in more detail by Romano et al. [48]. The NanoDropOne spectrophotometer (Thermo Scientific, Waltham, MA, USA) and the Qubit Fluorometer 4.0 (Invitrogen Co., Carlsbad, CA Briefly, USA) were both utilized to evaluate DNA quantity and quality. Then, PCR amplification was applied to the hyper-variable V3 and V4 regions of the 16S rRNA gene using the following primers: Forward 5′-CCTACGGGNGGCWGCAG-3′ and Reverse 5′-GACTACHVGGG TATCTAATCC-3′ [49]. A 16S Metagenomic Sequencing Library Preparation (Illumina, San Diego, CA) was used to collect each PCR reaction. Qubit fluorometer (Invitrogen Co., Carlsbad, CA, USA) was then used to quantify the identified libraries, which were pooled to an equimolar quantity of each index-tagged sample up to a 4 nM concentration. MiSeq platform (Illumina, San Diego, CA, USA) was used to obtain sequences from pooled samples in a 2 × 250 paired-end format. The FASTQ software was finally used for the quality control of the generated raw sequence files. The absence of contamination was ensured by a negative control consisting of all the reagents but the DNA template and used over each sample processing. The 16S Metagenomics app (Illumina, San Diego, CA, USA, Version 1.1.0), a high-performance procedure based on the Ribosomal Database Project (RDP) classifier [50], was used to perform the taxonomic classification of amplicon 16S rRNA-gene reads. The RefSeq RDP 16S v3 May 2018 DADA2 32bp [51] represented the used taxonomic database.

### 2.4. Statistical Techniques for Compositional Data Analysis

We first selected the 30 genera and the 30 species with the highest number of reads in each sample. Then, we selected the ones common in at least 50% of the 17 samples. In this way, we obtained 25 genera and 20 species to apply the CoDa analysis approach described in the following. The CLR (centered log-ratio) transformation of the selected bacterial genus and species reads was first performed. Note that the CLR ratio transformations produce the same results whether the data are counts or proportions (i.e., their relative abundances) and make the data symmetrical and linearly related in a log-ratio coordinate space (e.g., [11]). If we consider an observation vector of N “counted” features (e.g., reads, OTUs, taxa, etc.) in a sample, denoted as x = [x_1_, x_2_,…, x_N_], the CLR transformation for that sample can be estimated by using the following formula:

x_CLR_ = [log(x_1_/G(x)), log(x_2_/G(x)), …, log(x_N_/G(x))] (1)

where G(x) represents the geometric mean of the observation vector x. In more detail, the CLR-transformed data present a fundamental property that makes them particularly relevant for the CoDa analysis approach: they are scale-invariant. This last property means that the same ratios can be obtained in a sample with few counts or an identical sample with many counts, while the only difference can be the precision of the CLR calculation. Note that the CLR-transformed matrix of the initial dataset cannot be determined without replacing each zero-count value, otherwise it would not be possible to calculate the x_CLR_ values for each sample because of the zero value of the denominator in (1). Several methodologies were developed for the zero-count replacement, as summarized by Lubbe et al. [52]. The most common technique is to replace zero counts with a constant value smaller than the detection limit. We have followed the methodology proposed by Martín-Fernandez et al. [53]. They found that 65% of the detection limit minimizes the distortion in the covariance structure. Therefore, since our detection limit is represented by 1 count, we replaced each zero count with 0.65 in our dataset. The centered log-ratio (CLR) transform was then applied to the zero-replaced datasets to produce heatmaps (we used the *heatmap* function that is natively provided in the R package). Then, we calculated the matrix of the Aitchison distances among the samples being appropriate for both clustering and ordination techniques [16,54]. The Aitchison distance matrix was used as an input parameter to calculate the corresponding dendrogram showing the relationships among the studied samples. In more detail, to plot the dendrograms, we used the unweighted pair-group average, which means that the dendrogram-clusters are joined based on the average distance between all members in each couple of groups. The exploratory data analysis to study the relationships among OTUs (bacterial genera or species) and samples was subsequently performed by using a principal component analysis (PCA). More specifically, we performed the compositional PCA of our datasets by a singular value decomposition (SVD) of the CLR-transformed data [2] using the *prcomp* function under the R package *stats*. The PCA via SVD allows producing the score and loading plots (based on the output matrices called *x* and *rotation* estimated by the *prcomp* R function, respectively) to further examine the correlations/covariances among samples and OTUs. As specified by Bian et al. [19], in the score plot, each point represents a sample, and the distance between two points is proportional to the corresponding multivariate difference between the samples. Conversely, the loading plot represents the contribution of the OTUs to the separation of the samples. 

The ρ metrics [26] were used to analyze the correlations among the investigated genera and among the investigated species. In more detail, the ρ metrics can be estimated by the following formula:

ρ (A_i_, A_j_) = 2 cov (A_i_, A_j_)/(var (A_i_) + var (A_j_)) (2)

where the parameters A_i_ and A_j_ represent log (*s* · X_i_) and log (*s* · X_j_), respectively, *s* is the total number of counts for a given sample, while X_i_ and X_j_ represent the number of counts for a specific bacterial genus (or species) i and j for all the analyzed samples, respectively, and cov and var are the covariance and variance, respectively. We estimated the ρ metrics by using the R package *propr*.

Finally, the Shannon and Simpson indices (*H* and *D*, respectively) were calculated to investigate richness and biodiversity of the investigated samples (e.g., [55,56]). The *H* and *D* parameters were evaluated by the following formulas:

*H* = −Σ*_i_ p_i_* ln *p_i_* (3)

*D* = Σ*_i_* (*p_i_*)^2^ (4)

where *p_i_* is equal to *n_i_*/*N*, with *n_i_* representing the number of individuals in the species *i* and *N* the relative total number in all the community [57]. Note that the larger the value of *H* is, the larger the diversity of species becomes, while species richness and evenness decrease if *D* increases. Therefore, *D* represents a parameter associated with the diversity that considers both the number of species in each community and the relative abundance of each species.

## 3. Results and Discussion

Main results both at the genus- and species-level are presented and discussed in this section to support the comparison with previous studies presenting results only at the genus- (e.g., [58]) or species-level (e.g., [59]). 

### 3.1. Centered Log-Ratio Heatmap of Selected Bacterial Genera and Within-Sample Alpha-Diversity

**Figure 1:**
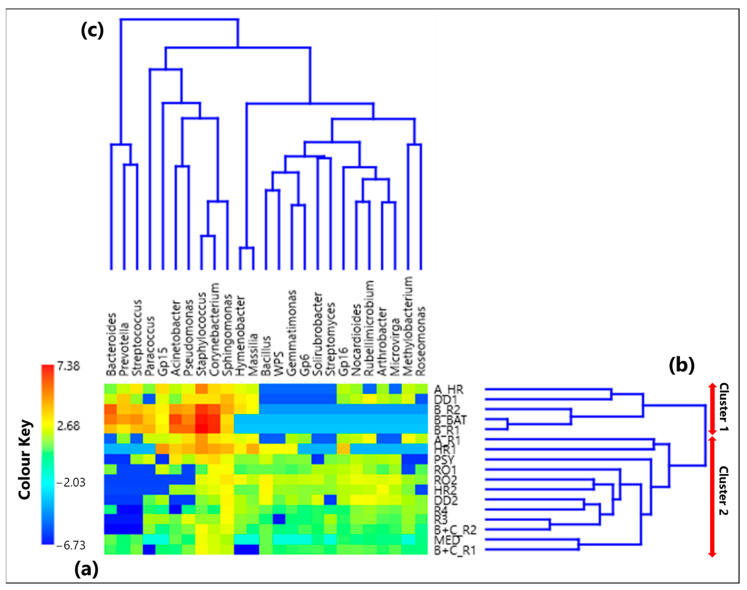
(**a**) Heatmap based on the centered log-ratio (CLR) transformed values of the 25 selected bacterial genera; (**b**,**c**) show the Aitchison distance-based dendrograms highlighting the relatedness between different samples and genera, respectively. The red arrows in (**b**) allow identifying the two main sample clusters identified by the dendrogram, where Cluster 1 includes the samples A_HR, DD1, B_R2, B_BAT, and B_R1, and Cluster 2 consists of all the other samples. WPS in the genus legend stands for WPSUnclassified1_genera_incertae_sedis.

The CoDa analysis approach has been applied to the 25 selected genera. Table S1 provides the CLR values associated with each genus in all the 17 samples, while Figure 1a displays by a color plot the CLR-heatmap of the 25 selected genera, which are listed in the figure in addition to the 17 samples where they were detected. Samples denoted according to Section 2.2 are listed in Table 1, as previously mentioned. The dendrograms of Figure 1b,c based on the Aitchison distances provide a preliminary indication on the association between samples and genera, respectively. The corresponding Aitchison matrix is reported in Table S2. The color plot of Figure 1a indicates that the CLR value associated with each genus varies among samples, because of the dependence of the sample taxonomic structure on the sampling location. Two main clusters of samples can be identified in Figure 1b: Cluster 1, which consists of the samples A_HR, DD1, B_R2, B_BAT, and B_R1, and Cluster 2, which consists of all the other samples. Note that Cluster 1 samples were all collected in rooms that hosted COVID-19 patients.

**Figure 2:**
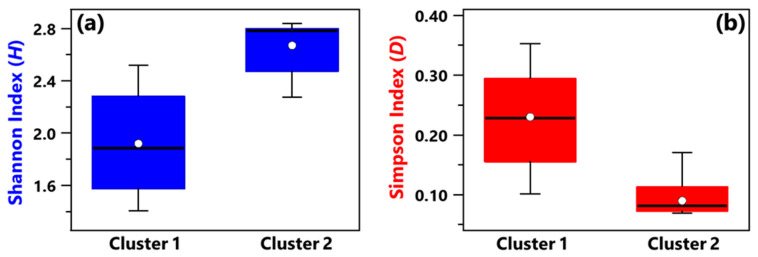
Boxplots displaying the (**a**) Shannon and (**b**) Simpson index value calculated at the genus level for the samples belonging to Cluster 1 (A_HR, DD1, B_R2, B_BAT, B_R1) and Cluster 2 (A_R1, HR1, PSY, RO1, RO2, HR2, DD2, R4, R3, B+C_R2, MED, B+C_R1). For each boxplot, the line within the box and the white dots represent the median and mean value, respectively. The bottom and top boundaries of each boxplot indicate the 25th and 75th percentiles, respectively. The whiskers are the 5th and 95th percentiles, respectively.

Shannon and Simpson indices of each sample have been evaluated to quantify the genera community alpha-diversity. Calculated values are reported in Table 1. *H* and *D* reach the highest and the smallest value, respectively, in sample R3, which was collected in a conventional room without any patients. Therefore, R3 is the sample with the largest diversity and richness/evenness at the genus-level. In contrast, *H* and *D* reach the smallest and the highest value, respectively, in sample B_R1, collected in a conventional room occupied by patient B. Consequently, sample B_R1 is characterized by the lowest diversity and richness/evenness at the genus-level. Figure 2a,b shows by box plots the Shannon and Simpson indices, respectively, which referred to all the samples associated with Cluster 1 and 2 from Figure 1b. The median and mean *H* values of the Cluster 1 box plot are smaller than the corresponding values of the Cluster 2 box plot (Figure 2a). Cluster 1 consists of samples collected in rooms all occupied by COVID-19 patients, while most of the samples collected in rooms without any COVID-19 patient contribute to Cluster 2. Therefore, Figure 2a shows that the Cluster 1 samples are on average characterized by smaller diversity at the genus-level than Cluster 2 samples. Figure 2b shows that the *D* median and mean values of the Cluster 1 box plot are higher than the corresponding values of the Cluster 2 box plot, because of the smaller richness/evenness of the samples collected in rooms occupied by COVID-19 patients. 

#### 3.1.1. Singular Value Decomposition PCA by Score and Loading Plots at the Genus-Level

**Figure 3:**
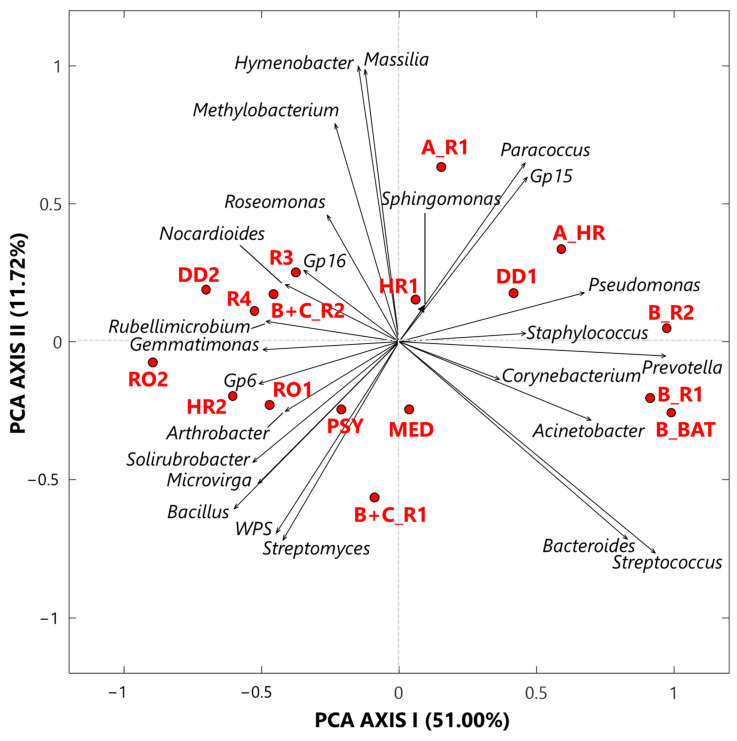
Two-dimensional principal component analysis biplot made via a singular value decomposition of the CLR-transformed values for the 25 selected bacterial genera. The reported biplot illustrates the relationships between samples (score plot, red dots) and genera (loading plot, black arrows). The percentages of the total variance explained by the first and second principal components are also reported. Note that *WPS* represents *WPSUnclassified1_genera_incertae_sedis*.

The exploratory analysis of the CLR-transformed genus dataset was also performed by the SVD-PCA to examine the relationships among samples and selected bacterial genera, according to Gloor et al. [2]. SVD-PCA outputs are driven by the genera with the largest variation in the dataset and allow identifying main relationships between samples and genera by comparing score and loading plots, which are shown in Figure 3 by red dots and black arrows, respectively. Note that in the loading plot of Figure 3, the distance from the origin and the direction of each arrow is proportional to the standard deviation of the associated genus CLR value in the investigated dataset [19]. Then, the distance between two arrows is inversely proportional to their compositional association: the closeness between two arrows implies that the associated genera may have concordant abundances within closest samples (red dots). We selected the SVD-PCA analysis since it represents the preferred methodology when the number of input bacterial genera is larger than the corresponding number of samples, as it occurs in our case, being the number of genera and samples equal to 25 and 17, respectively. The variance percentage explained by the first and second synthetic PCA axis, which is equal to 51.00% and 11.72%, respectively, highlights a good performance of the used technique. The score plot (dots) in Figure 3 firstly shows that all samples associated with Cluster 1 are on the right side of the PCA-Axis 1, while most of the samples associated with Cluster 2 are on the left side of the PCA-Axis 1. Sample A_R1 on the upper right-side quarter and sample B+C_R1 on the lower left-side quarter of Figure 3, which are Cluster 2 samples collected in rooms with COVID-19 patients, are the extreme ones, likely because of their different bacterial structure with respect to that of the other Cluster 2 samples. The color plot in Figure 1a supports the last comment. The loading (arrows) plot in Figure 3 highlights the rather different bacterial structure between Cluster 1 and Cluster 2 samples. *Pseudomonas, Staphylococcus*, *Prevotella*, *Corynebacterium*, and *Acinetobacter* are mainly associated with samples B_R2, B_R1, and B_BAT, which were all collected in rooms that hosted patient B, and with samples DD1 and A_HR. In contrast, *Sphingomonas*, *Paracoccus,* and *Gp15* are mainly associated with samples HR1 and A_R1. *Corynebacterium*, *Sphingomonas*, and *Staphylococcus* are the only three genera detected in all the samples with high and positive CLR values, as reported in Table S1. In fact, they are among the prevailing genera within hospital wards (e.g., [60,61]). *Corynebacterium* is a skin colonizer that also has been isolated in patients after prolonged hospitalization (e.g., [61,62]). *Sphingomonas* may be responsible for some nosocomial infections from environmental exposure [62]. The genus *Staphylococcus* includes more than 45 species, mostly commensals of the skin and mucous surfaces of humans and other mammals, and it is responsible for infections especially in patients undergoing prolonged hospitalization and/or antibiotic therapy, or with comorbidities (e.g., [63]). Ribeiro et al. [58] used some deep-sequencing analyses to examine and compare the structure of bacterial communities in the intensive care units (ICU) and neonatal intensive care units (NICU) of The Medical School Clinics Hospital in Brazil. They found that *Staphylococcus* was one of the most abundant genera both in ICU and in NICU, in agreement with previous studies [64,65,66]. *Staphylococcus* can present large abundances in ICU likely because it can persist for months on dry surfaces [67] and/or can be associated with spore or biofilm formation [68]. *Acinetobacter* and *Pseudomonas,* which are two among the five genera mainly associated with the three samples collected in the patient B’s room, also represent two of the most common pathogens causing hospital-associated infections (HAIs), according to Magill et al. [69]. Moreover, Ribeiro et al. [58] specified that these genera can be usually found in moist environments and may imply a high risk of HAI in immunocompromised patients. *Prevotella* also represents a potentially dangerous human pathogen, and it was found with high abundances on the surfaces near hospital computers (more specifically near keyboard and mouse), according to Ribeiro et al. [58]. *Bacteroides* and *Streptococcus* genera reached high and positive CLR values in the samples B_R2, B_R1, and B_BAT (Figure 1a). *Bacteroides* is a common anaerobe that occupies the intestines of humans. It is also the most common anaerobe recovered from various infections, such as intra-abdominal infection, foot ulcer, and bloodstream infection (e.g., [70]). In more detail, the *Bacteroides fragilis* group represents one of the most important anaerobic clinical pathogens and ranges under the 15 most common pathogens causing nosocomial infections (e.g., [71]). Within the *Streptococcus* genus, the related species *S. pneumoniae* is a major cause of community-acquired pneumonia, bacteraemia, and meningitis, with asymptomatic nasopharyngeal colonization generally representing a predisposing factor for pneumococcal infections [72]. 

The sample MED (Figure 3) cannot be significantly associated with any specific bacterial genera. In fact, it was collected in the medicine-store room, which was a less contaminated place, and consequently presents low or negative CLR values for almost all the investigated bacterial genera (Figure 1a). As mentioned, the loading plot of Figure 3 shows that the bacterial structure of all Cluster 2 samples is rather different from that of the Cluster 1 samples, as Cluster 2 samples were mainly collected in rooms without any COVID-19 patient. Consequently, the loading plot on the left-side of Figure 3 shows both that the bacterial structure varies among Cluster 2 samples and that most genera are non-pathogenic. As an example, *Gp16*, *Nocardioides*, and *Rubellimicrobium*, which are mainly associated with the Cluster 2 samples R3, DD2, R4, and B+C_R2 (Figure 3), are non-pathogenic genera isolated from soil, while *Roseomonas,* which also reached positive CLR values in the above samples (Table S1), is a pathogenic genus associated with bacteraemia and other human infections [73]. *Arthrobacter* and *Solirubrobacter*, which are mostly associated with samples HR2 and RO1, also are genera commonly detected in soil [74,75]. *Bacillus, Microvirga,* and *Streptomyces*, which are associated with the PSY sample, in addition to *WPS* and *Solirubrobacter*, also are widely found in soil.

#### 3.1.2. Proportionality between Genera by the ρ Metrics

Table 2 summarizes the significant ρ values among the selected 25 genera, with both positive and negative values (larger than 0.65 and lower than −0.65, respectively) reported in brackets. The complete ρ matrix is displayed in Table S3. Few significant positive ρ values among a pair of pathogenic genera associated with Cluster 1 samples have been detected. More specifically, Table 2 displays positive and significant ρ values among *Corynebacterium* and *Staphylococcus* (0.92), *Acinetobacter* and *Pseudomonas* (0.77), *Bacteroides* and *Prevotella* (0.78), *Bacteroides* and *Streptococcus* (0.76), and *Prevotella* and *Streptococcus* (0.83). The comparison of these results with the hierarchical clustering of clades on the left-side of Figure 1c shows that the detected significant proportionalities between the abovementioned pairs of pathogenic genera correspond to double clades in the dendrogram of Figure 1c. Moreover, these pathogens may all cause hospital-associated infections, as discussed in the previous section (e.g., [58,61,69,76,77,78,79]). The high proportionalities found among *Bacteroides*, *Prevotella,* and *Streptococcus* can also be due to the fact that they are commonly found in the upper respiratory tract (e.g., [80,81]). Table 2 also shows that *Corynebacterium* is characterized by significant negative ρ values with *Nocardioides* (−0.78), *Arthrobacter* (−0.66), and *Rubellimicrobium* (−0.66), which are non-pathogenic genera mainly isolated from soil. *Staphylococcus* is characterized by significant negative ρ values with *Microvirga* (−0.79), *Gp6* (−0.66), and *Solirubrobacter* (−0.67), in addition to *Nocardioides* (−0.78), *Arthrobacter* (−0.74), and *Rubellimicrobium* (−0.75).

Significant positive ρ metrics values have also been found among the non-pathogenic bacteria located at the left-side of Figure 3. In fact, Table 2 displays a significant positive ρ value between *Hymenobacter* and *Massilia* (0.98), which can be mainly associated with the samples A_R1 and HR1, respectively. Both genera were isolated in soil samples (e.g., [82,83,84,85]), and Samaké et al. [86] identified them as two of the most abundant bacterial genera in PM10 samples collected in a rural background site in France. Significant positive ρ values also occur among *Bacillus* and *Gemmatimonas* (0.78), *Microvirga* (0.69), *Gp6* (0.66), *Solirubrobacter* (0.71), *WPS* (0.85), and *Streptomyces* (0.66), which form a cluster of nested clades on the right side of the dendrogram in Figure 1c. 

In conclusion, Table 2 has shown that significant positive ρ values between two genera have a close correspondence with the double clades from the Aitchison distance-based dendrogram in Figure 1c, which also highlights the relatedness between different genera.

### 3.2. Centered Log-Ratio Heatmap of Selected Bacterial Species and Within-Sample Alpha-Diversity

**Figure 4:**
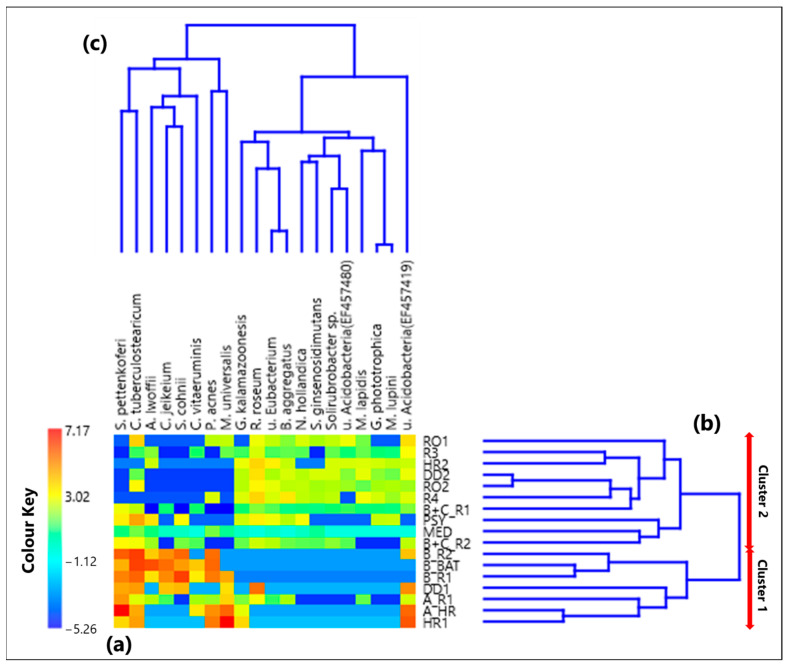
(**a**) Heatmap based on the centered log-ratio (CLR) transformed values of the 20 selected bacterial species reads; (**b**,**c**) show the Aitchison distance-based dendrograms highlighting the relatedness between different samples and species, respectively. The two main sample clusters defined by the corresponding dendrogram are also indicated in (**b**). Legend: *S.* (*Staphylococcus*) *pettenkoferi*, *C.* (*Corynebacterium*) *tuberculostearicum*, *A.* (*Acinetobacter*) *lwoffii*, *C.* (*Corynebacterium*) *jeikeium*, *S.* (*Staphylococcus*) *cohnii*, *C.* (*Corynebacterium*) *vitaeruminis*, *P.* (*Propionibacterium*) *acnes*, *M.* (*Methyloversatilis*) *universalis*, *G.* (*Gemmatirosa*) *kalamazoonesis*, *R.* (*Rubellimicrobium*) *roseum*, *u.* (*uncultured*) *Eubacterium*, *B.* (*Blastococcus*) *aggregatus*, *N.* (*Nitrolancea*) *hollandica*, *S.* (*Solirubrobacter*) *ginsenosidimutans*, *u.* (*uncultured*) *Acidobacteria* (EF457480), *M.* (*Modestobacter*) *lapidis*, *G.* (*Gemmatimonas*) *phototrophica*, *M.* (*Microvirga*) *lupini*, and *u.* (*uncultured*) *Acidobacteria* (EF457419).

The heatmap based on the centered log-ratio values of the 20 selected species is shown by a color-plot in Figure 4a, while the within-sample CLR-values for each species are in Table S4. Figure 4b,c show the Aitchison distance-based dendrograms. Table S5 provides the corresponding Aitchison-distance matrix. The red arrows on the right side of Figure 4b allow identifying two main cluster of samples. Cluster 1 consists of the samples B_R2, B_BAT, B_R1, DD1, A_R1, A_HR, and HR1, which were all collected in rooms that hosted COVID-19 patients, except for sample HR1, which was collected in an empty high isolation room. All the other 10 samples contribute to Cluster 2, collected in rooms without any patients, except for B+C_R1, B+C_R2, and PSY samples.

**Figure 5:**
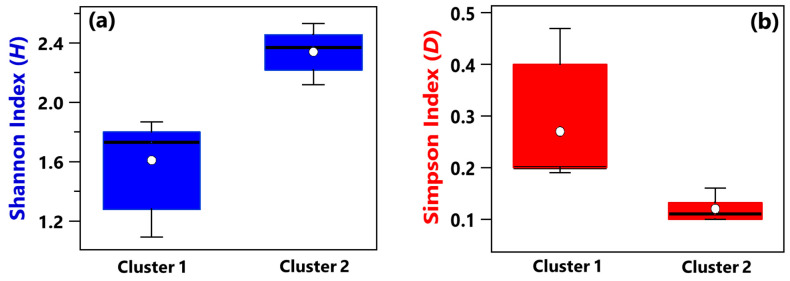
Boxplots displaying (**a**) Shannon and (**b**) Simpson indices calculated at the species level for samples belonging to Cluster 1 (B_R2, B_BAT, B_R1, DD1, A_R1, A_HR, HR1) and Cluster 2 (RO1, R3, HR2, DD2, RO2, R4, B+C1_R1, PSY, MED, B+C_R2). For each boxplot, the line within the box and the white dots represent the median and mean value, respectively. The bottom and top boundaries of each boxplot indicate the 25th and 75th percentiles, respectively. The whiskers are the 5th and 95th percentiles, respectively.

Alpha-diversity of the bacterial species community was investigated by means of Shannon (*H*) and Simpson (*D*) indices, whose values computed for each of the 17 samples are listed in Table 1. The sample B+C_R1, collected in a conventional room hosting the COVID-19 patients B and C, is characterized by the highest *H* value and the lowest *D* value; therefore, it is the sample with the greatest species diversity and richness/evenness. On the contrary, *H* and *D* reached the smallest and the largest value, respectively, in the HR1 sample collected in a high isolation room with no patients. Figure 5a,b show by box plots the Shannon and Simpson indices, respectively, referred to all the samples associated with Clusters 1 and 2 from Figure 4b. The median and mean *H* values of the Cluster 1 box plot are smaller than the corresponding values of the Cluster 2 box plot (Figure 5a). In contrast, the median and mean *D* values of the Cluster 1 box plot are greater than the corresponding values of the Cluster 2 box plot (Figure 5b), similarly to the findings at the genus level. Therefore, Cluster 1 samples are on average characterized by a smaller diversity and richness/evenness than Cluster 2 samples at the species level. 

#### 3.2.1. Singular Value Decomposition PCA by Score and Loading Plots at the Species Level

**Figure 6:**
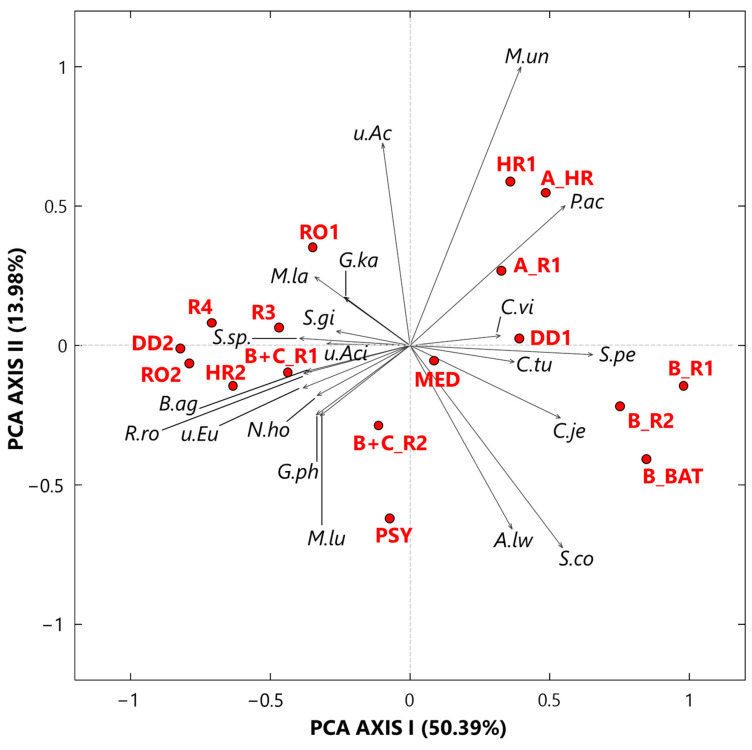
Two-dimensional SVD-PCA of the CLR-transformed values for the 20 selected bacterial species by the score (red dots) and the loading (black arrows) plot. The percentages of the total variance explained by the first and second principal components are also reported. Legend: *M.un* (*Methyloversatilis universalis*), *P.ac* (*Propionibacterium acnes*), *C.vi* (*Corynebacterium vitaeruminis*), *S.pe* (*Staphylococcus pettenkoferi*), *C.tu* (*Corynebacterium tuberculostearicum*), *C.je* (*Corynebacterium jeikeium*), *S.co* (*Staphylococcus cohnii*), *A.lw* (*Acinetobacter lwoffii*), *M.lu* (*Microvirga lupini*), *G.ph* (*Gemmatimonas phototrophica*), *N.ho* (*Nitrolancea hollandica*), *u.Eu* (*uncultured Eubacterium*), *R.ro* (*Rubellimicrobium roseum*), *B.ag* (*Blastococcus aggregatus*), *u.Aci* (*uncultured Acidobacteria* EF457480), *S.*sp. (*Solirubrobacter* sp.), *S.gi* (*Solirubrobacter ginsenosidimutans*), *G.ka* (*Gemmatirosa kalamazoonesis*), *M.la* (*Modestobacter lapidis*), and *u.Ac* (*uncultured Acidobacteria* EF457419).

The SVD-PCA was applied to the CLR-transformed bacterial species dataset to explore how the 17 samples and the 20 selected species are potentially linked to each other. Figure 6 displays the PCA score (red dots) and loading (black arrows) plots. The variance percentages explained by the first and second synthetic PCA axes are equal to 50.39% and 13.98%, respectively, which implies a good performance of the applied technique. All the Cluster 1 samples, in addition to sample MED, are located on the right-side half plane of Figure 6 SVD-PCA biplot, while all the other samples are on the left-side half plane of Figure 6. Except HR1 collected in a high isolation room with no patients, all the other Cluster 1 samples were collected in rooms with COVID-19 patients. *Propionibacterium acnes*, *Corynebacterium vitaeruminis*, *Staphylococcus pettenkoferi*, *Corynebacterium tuberculostearicum*, and *Corynebacterium jeikeium* are the main species associated with the samples collected in rooms with COVID-19 patients. *Propionibacterium acnes* is a microaerophilic and gram-positive bacterium that resides in the pilosebaceous follicles of the human skin [87]. It is a low-virulence opportunistic pathogen, which may cause a wide range of infections, often following surgery and in relation with the use of medical devices [88]. Cases of elbow joint and prosthetic joint infections, as well as post-operative discitis, have also been reported [89,90,91,92]. *Corynebacterium vitaeruminis* has already been proved to be safe and non-pathogenic; indeed, this strain was negative for 50 tested virulence and resistance genes based on performed PCR, according to Colombo et al. [93]. Nevertheless, its genus includes numerous species, which are increasingly recognized as important pathogens related to human and animal diseases [94]. *Staphylococcus pettenkoferi*, originally described in Germany in 2002 by Trülzsch et al. [95], is part of a group of bacteria known as *Coagulase-Negative staphylococci* (*CoNS*), which are typical skin flora but potentially portend pathogenicity against humans. A Canadian literature review [96] describes nine case reports of *S. pettenkoferi* true bacteraemia worldwide [97]. This has been attributed not only to nosocomial acquisition from the increased use of intravascular catheters and cardiac devices but also to the aging of the population, as well as the larger number of immunocompromised patients [98]. *Corynebacterium tuberculostearicum* and *Corynebacterium jeikeium*, in addition to *Staphylococcus cohnii* and *Acinetobacter lwoffii*, are ubiquitous species in the environment (soil and water) and often commensal of normal human skin and mucous membranes [99]. More specifically, *C. tuberculostearicum* has been found as a frequent colonizer on the skin of hospitalized patients, causing or not causing infections [100], while *C. jeikeium* has frequently been isolated from clinical specimens and has demonstrated nosocomial transmission. *Acinetobacter lwoffii* is a potential opportunistic pathogen isolated in immunocompromised patients and has been considered to play a role in nosocomial infections, such as septicaemia, meningitis, and pneumonia [101]. Moreover, *Staphylococcus cohnii* is a common *CoNS* species frequently detected in hospital wards and characterized by a notable antibiotic resistance [102]. 

All the bacterial species associated with Cluster 2 samples are located on the left half-plane of Figure 6, and they are mostly non-pathogenic. *Rubellimicrobium roseum*, as an example, reached one of the highest CLR-value in sample DD2 (Table S4) and was reported as one of the most ubiquitous soil and organic material-dwelling bacteria in outdoor PM [103].

The sample MED (Figure 6) is close to the origin of the PCA axis as in Figure 3, because it was collected in an aseptic area, and consequently, it is not associated with any bacterial species. In conclusion, the SVD PCA biplot has clearly proved that the relationships among samples and bacterial species depend strongly on monitoring locations. 

#### 3.2.2. Proportionality between Species by the ρ Metrics

Table 3 summarizes the significant positive and negative ρ metrics values among the selected 20 species, where significant values (larger than 0.65 and lower than −0.65) have been reported in brackets. The complete ρ matrix is reported in Table S6. Rather few significant positive ρ values between pairs of pathogenic species associated with Cluster 1 samples have been detected. More specifically, a high proportionality was identified between *S. pettenkoferi* and *C. jeikeium* (0.66) and between *C. jeikeium* and *S. cohnii* (0.68), which were mainly associated with the samples collected in the rooms that hosted patient B (Figure 6). 

Table 3 shows high estimated ρ values also among *R. roseum*, *Uncultured eubacterium*, *B. aggregatus*, *N. hollandica*, and *Solirubrobacter* sp., which are species mainly associated with Cluster 2 samples. Moreover, similarly to what was found about bacterial genera (see Section 3.1.2), Table 3 shows that all the significant negative ρ metrics (<−0.65) concern the relationship between bacterial species belonging to Cluster 1 and those belonging to Cluster 2, likely because most of the Cluster 1 samples have been collected in rooms that hosted COVID-19 patients. 

## 4. Conclusions

The 16S rRNA gene sequencing dataset from airborne samples collected mainly at the Infectious Disease (ID) Department at Santa Caterina Novella Hospital in Galatina (Lecce, Italy) has been investigated in this study by means of a compositional data approach. Exploring the airborne bacterial community structure within a nosocomial area, which hosted COVID-19 patients, determining the impact of the sampling location on the bacterial taxonomy, and investigating the relationship among taxa represented the main goals of the current paper.

- Eight and six samples were collected at the ID ward, in rooms with and without COVID-19 patients, respectively. Moreover, a sample (PSY) collected at the psychiatry department and two outdoor samples (RO1 and RO2) collected on the roof of the ID department were also analyzed to compare their bacterial profiles with the corresponding ones of indoor samples from the ID ward.
- Twenty-five genera, selected from the ones reaching the largest read number in each sample and common to at least 50% of the 17 collected samples, were analyzed by the compositional approach. More specifically, the SVD-PCA applied to CLR dataset has been used to investigate the relationship among collected samples and selected bacterial genera.
- The SVD-PCA score plot has shown that all samples could be divided in two groups: Cluster 1, mainly consisting of samples collected in rooms occupied by COVID-19 patients, and Cluster 2, which included samples mostly collected in rooms without any COVID-19 patients, as well as outdoor samples.
- The SVD-PCA loading plot has highlighted the different genus structure associated with the samples of Cluster 1 and 2, respectively. *Sphingomonas*, *Paracoccus, Gp15, Pseudomonas, Staphylococcus*, *Prevotella*, *Corynebacterium*, and *Acinetobacter* genera were mainly associated with Cluster 1 samples, and they can be responsible for different types of nosocomial infections.
- In contrast, *Gp16*, *Nocardioides*, *Rubellimicrobium*, *Arthrobacter,* and *Solirubrobacter* were among the non-pathogenic genera isolated from soil and associated with Cluster 2 samples.
- Shannon and Simpson indices calculated at the genus level have shown that, on average, Cluster 1 samples were characterized by smaller diversity and richness/evenness than Cluster 2 samples.
- The ρ metrics showed few significant positive values between genera associated with Cluster 1 samples. More specifically, positive significant ρ values were found between *Corynebacterium* and *Staphylococcus* (0.92), *Acinetobacter* and *Pseudomonas* (0.77), *Bacteroides* and *Prevotella* (0.78), *Bacteroides* and *Streptococcus* (0.76), and *Prevotella* and *Streptococcus* (0.83). Moreover, it has been found that *Corynebacterium* and *Staphylococcus* were characterized by significantly negative ρ proportionality with some non-pathogenic genera associated with Cluster 2 samples.
- Significant positive ρ metrics values have also been found among some non-pathogenic bacteria associated with the Cluster 2 samples, as the ones between *Hymenobacter* and *Massilia* (0.98), and *Bacillus* and *Gemmatimonas* (0.78), as well as *Microvirga* (0.69), *Gp6* (0.66), *Solirubrobacter* (0.71), *WPS* (0.85), and *Streptomyces* (0.66).
- Twenty bacterial species were also selected and analyzed by the SVD-PCA applied to the CLR-transformed species dataset. Then, the score and loading plots allowed dividing all samples into two clusters characterized by different bacterial species.
- Cluster 1 included all the samples collected in rooms with COVID-19 patients A and B, while Cluster 2 was mostly consisted of samples collected in rooms without COVID-19 patients. *Propionibacterium acnes*, *Corynebacterium vitaeruminis*, *Staphylococcus pettenkoferi*, *Corynebacterium tuberculostearicum*, and *Corynebacterium jeikeium* were the main species associated with Cluster 1 samples. Except for *Corynebacterium vitaeruminis*, which has been proved to be safe and non-pathogenic, all the other detected species have frequently been identified in hospitals as agents of nosocomial infections.
- Non-pathogenic species were mainly associated with Cluster 2 samples, such as *Rubellimicrobium roseum*, which was reported as one of the most ubiquitous soil and organic material-dwelling bacteria in outdoor particulate matter.
- Shannon and Simpson index mean values associated with Cluster 1 samples also featured a smaller diversity and richness/evenness than Cluster 2 samples.
- The ρ metrics also revealed strong proportionality between bacterial species of Cluster 1 samples, while negative relationships were found with non-pathogenic species detected in Cluster 2.

In conclusion, the compositional data approach applied to a 16S-rRNA-gene sequencing dataset to investigate the typical airborne microbiome within an infectious disease department, focusing on bacterial genera and species, has been discussed. Consistently with previous works, we found several genera and species commonly associated with nosocomial pathologies, mostly in samples collected in rooms hosting COVID-19 patients, while non-pathogenic taxa were mainly detected in samples collected in the absence of patients, as well as in outdoor samples. The impact of the sampling location on the detected bacterial distribution, both at the genus and species level, has been clearly demonstrated. Nevertheless, we are aware that the limited number of the analyzed samples may represent a disadvantage to this study, but bureaucratic and technical reasons did not allow us to perform additional samplings.

## Figures and Tables

**Table 1:** Alpha-diversity Shannon (*H*) and Simpson (*D*) indices calculated at the genus and species level for the 17 analyszed samples. The sampling date of each sample is also reported.

Sample	Date (dd/mm/yy)	At the Genus Level		At the Species Level	
		Shannon Index (*H*)	Simpson Index (*D*)	Shannon Index (*H*)	Simpson Index (*D*)
A_HR	30/04/20	2.05	0.24	1.28	0.40
A_R1	01/05/20	2.29	0.17	1.87	0.26
B_R1	05/05/20	1.41	0.35	1.79	0.20
B_R2	07/05/20	1.89	0.21	1.72	0.20
B_BAT	06/05/20	1.74	0.23	1.73	0.20
B+C_R1	17/05/20	2.83	0.08	2.53	0.10
B+C_R2	21/05/20	2.79	0.08	2.45	0.10
HR1	01/05/20	2.27	0.12	1.09	0.47
HR2	15/05/20	2.78	0.07	2.27	0.12
R3	02/05/20	2.84	0.07	2.48	0.11
R4	04/06/20	2.80	0.09	2.34	0.11
MED	03/05/20	2.43	0.12	2.15	0.14
RO1	08/05/20	2.79	0.08	2.24	0.13
RO2	16/07/20	2.78	0.07	2.44	0.10
PSY	11/07/20	2.60	0.10	2.12	0.16
* DD1	07/05/20	2.52	0.10	1.80	0.19
* DD2	07/05/20	2.78	0.07	2.40	0.11

* The date represents the starting sampling date since both samples were collected by dry deposition over 14 days.

**Table 2:** ρ metrics values among the selected 25 genera. Only significant positive (ρ values > 0.65) and negative (ρ values < −0.65) values are reported in the table. *WPS* in the “Bacterial Genera” column represents *WPS Unclassified1_genera_incertae_sedis*.

Bacterial Genera	Positive Correlations	Negative Correlations
*Corynebacterium*	*Staphylococcus* (0.92)	*Nocardioides* (−0.78), *Arthrobacter* (−0.66), *Rubellimicrobium* (−0.66)
*Staphylococcus*		*Nocardioides* (−0.78), *Arthrobacter* (−0.74), *Rubellimicrobium* (−0.75), *Microvirga* (−0.79), *Gp6* (−0.66), *Solirubrobacter* (−0.67)
*Acinetobacter*	*Pseudomonas* (0.77)	
*Pseudomonas*		*Solirubrobacter* (−0.70)
*Hymenobacter*	*Massilia* (0.98)	
*Nocardioides*	*Arthrobacter* (0.75), *Rubellimicrobium* (0.79)	
*Arthrobacter*	*Microvirga* (0.82)	
*Rubellimicrobium*	*Microvirga* (0.75), *Gp6* (0.73)	
*Bacillus*	*Gemmatimonas* (0.78), *Microvirga* (0.69), *Gp6* (0.66), *Solirubrobacter* (0.71), *WPS* (0.85), *Streptomyces* (0.66)	*Prevotella* (−0.78)
*Gemmatimonas*	*Gp6* (0.83), *WPS* (0.68)	*Bacteroides* (−0.66), *Prevotella* (−0.68), *Streptococcus* (−0.67)
*Bacteroides*	*Prevotella* (0.78), *Streptococcus* (0.76)	
*Prevotella*	*Streptococcus* (0.83)	

**Table 3:** Relationships among the 20 selected bacterial species based on the ρ metrics proportionality. Only significant positive (ρ values > 0.65) and negative (ρ values < −0.65) values are reported in the table.

Bacterial Species	Positive Correlations	Negative Correlations
*Corynebacterium tuberculostearicum*		*Uncultured eubacterium* (−0.66), *Blastococcus aggregatus* (−0.69), *Modestobacter lapidis* (−0.72), *Solirubrobacter* sp. (−0.66)
*Rubellimicrobium roseum*	*Uncultured eubacterium* (0.75), *Blastococcus aggregatus* (0.76)	*Propionibacterium acnes* (−0.71)
*Staphylococcus pettenkoferi*	*Corynebacterium jeikeium* (0.66)	*Modestobacter lapidis* (−0.71), *Solirubrobacter* sp. (−0.76)
*Uncultured eubacterium*	*Blastococcus aggregatus* (0.98), *Nitrolancea hollandica* (0.67), *Solirubrobacter* sp. (0.68)	*Corynebacterium jeikeium* (−0.69)
*Blastococcus aggregatus*	*Modestobacter lapidis* (0.68)	*Corynebacterium jeikeium* (−0.67)
*Nitrolancea hollandica*	*Solirubrobacter ginsenosidimutans* (0.66)	
*Modestobacter lapidis*		*Staphylococcus cohnii* (−0.73)
*Solirubrobacter sp.*	*Solirubrobacter ginsenosidimutans* (0.69), *uncultured Acidobacteria(EF457480)* (0.83)	
*Corynebacterium jeikeium*	*Staphylococcus cohnii* (0.68)	
*Gemmatimonas phototrophica*	*Microvirga lupini* (0.99)	

## Data Availability

Data is contained within the article or Supplementary Material.

## Supplementary Materials

The following supporting information can be downloaded at: https://www.mdpi.com/article/10.3390/ijerph191610107/s1, Figure S1: Schematic picture of the intensive care unit; Table S1: Heatmap of the centered log-ratio (CLR) values of the 25 bacterial genera; Table S2: Aitchison distance matrix of within-sample CLR values associated with the 25 bacterial genera; Table S3: Matrix based on ρ metrics among the 25 bacterial genera; Table S4: Heatmap of the CLR values of the 20 bacterial species; Table S5: Aitchison distance matrix of within-sample CLR values associated with the 20 bacterial species; Table S6: Matrix based on ρ metrics among the 20 bacterial species.
